# Intestinal Parasites in Children from a Day Care Centre in Matanzas City, Cuba

**DOI:** 10.1371/journal.pone.0051394

**Published:** 2012-12-07

**Authors:** Roberto Cañete, Mariuska Morales Díaz, Roxana Avalos García, Pedro Miguel Laúd Martinez, Félix Manuel Ponce

**Affiliations:** 1 Centre for Hygiene, Epidemiology and Microbiology, Matanzas City, Cuba; 2 Faustino Pérez Hernández Hospital, Matanzas, Cuba; 3 Cuban Institute of Gastroenterology, Havana City, Cuba; Institut national de la santé et de la recherche médicale - Institut Cochin, France

## Abstract

**Background:**

Intestinal parasitic infections are widely distributed throughout the world and children are the most affected population. Day care centres are environments where children have proven to be more susceptible to acquiring IP.

**Methods and Principal Findings:**

A cross-sectional study was carried to determine the prevalence of intestinal parasites in stool samples among children who attend to a day care centre in an urban area of Matanzas city, Cuba, from March to June 2012. 104 children under five years old were included on the study after informed consent form was signed by parents or legal guardians. Three fresh faecal samples were collected from each child in different days and were examined by direct wet mount, formalin-ether, and Kato- Katz techniques. Data relating to demography, socioeconomic status, source of drinking water, and personal hygiene habits were also collected using a standardized questionnaire. In total, 71.1% of children harbored at least one type of intestinal parasite and 47 (45.2%) were infected by more than one species. *Giardia duodenalis* and *Blastocystis sp.* were the most common parasites found, with prevalence rates of 54.8% and 38.5% respectively.

**Conclusions:**

Despite public health campaigns, improvement in the level of education, and the availability of and access to medical services in Cuba infections by intestinal protozoan is high in this centre. Almost nothing is published regarding intestinal parasites in Matanzas province during the last 40 years so this work could also be the initial point to carry out other studies to clarify the IP status in this region.

## Introduction

Intestinal parasitic infections are widely distributed throughout the world and have been identified as one of the most significant causes of illnesses and diseases among the disadvantaged population [1].

It is estimated that 3 billion people are infected with intestinal parasites (IP) and children are the most common affected population [2]. In this group intestinal parasites can have devastating consequences affecting intestinal absorption, nutrition, and childhood development. While those infections are seen in developing countries, they are especially a threat to those living in developed countries because of traveling, trade and migration [3].

Day care centres are environments where children have proven to be more susceptible to acquiring IP. This observation agrees with the results reported by Gonçalves [3] and Menezes [4] in Brazil in 2011 and 2008, respectively. In Cuba, despite of the implementation of government initiatives to improve socioeconomic conditions, health, sanitation and water supplies [5], some parasitic infections, particularly intestinal protozoa, are still important causes of morbidity. This is particularly so in some high-risk groups, such as children attending day care, and preschool children in rural mountainous areas [6]–[9].

The aim of this study was to determine the prevalence of IP in stool samples from children who attend to a day care centre in an urban area of Matanzas city, Cuba.

## Methods

A cross-sectional study was carried out from March to June 2012 by researchers form the Department of Gastroenterology at Faustino Pérez Hernández Hospital and the Centre of Hygiene, Epidemiology and Microbiology, Matanzas city- Cuba.

### Study Population

104 children (the total population of the centre) from six months to five years of age, who attended to *Amiguitos de Nicaragua* day care centre, were studied. Parents or legal guardians signed the free informed consent form agreeing to the participation of the children. The institution belongs to an urban area of the city.

### Collection of Faecal Samples

Three faecal samples from each child were collected in a wide mouth screw capped containers free of preservative at intervals of two days. The samples were collected by parents, relatives or by the educator and immediately sent to the Department of Gastroenterology at Faustino Pérez Hernández Hospital.

Three slides of each sample were prepared and examined by two analysts (one technician and one medical parasitologist); such that six slides per sample were analyzed. Three fresh faecal samples were collected from each child in different days and were examined by direct wet mount and formalin-ether (Ritchie) techniques. Kato-Katz was used when analyzing the first sample of each child. The intensity of each STH infection was expressed as the mean of eggs per gram counts of the sample.

### Community Return

All families and educators received the results of the laboratory diagnosis. The positive cases were referred to appropriate healthcare units, where they received specific treatment and follow-up.

### Data Collection

A questionnaire was administered by researchers to each parent or legal guardian seeking for demography, socioeconomic status, source of drinking water, and personal hygiene habits which will be used to assess the potential risk factors for IPIs.

All children were apparently in good health condition and had no history of medication one month before the study commencement.

### Data Management and Statistical Analysis

Data derived from questionnaires and parasitological examinations were analyzed using EpiInfo 6.0 software (Public Health Domain Software, CDC, Atlanta, GA, USA). Initial data entry was cross-checked by two independent individuals in order to be sure that data were entered correctly. Prevalence was determined on the basis of combined results from the different diagnostic methods. For descriptive data, rate (percentage) was used to describe the characteristics of the studied group, including the prevalence of intestinal parasites according to age and gender.

A Pearson's Chi-square (*X^2^*) on proportion was used to test the associations between each variable. In univariate statistical model, the dependent variable was prevalence of intestinal parasites, while the independent variables were sociodemographic, behavior, environmental sanitation and living condition characteristics. A significant level of *p*<0.05 was adopted, Relative Risk (RR) and 95% confidence interval was computed by the analysis.

### Ethical Considerations

Ethical clearance was granted by the Research and Ethics Committee of Faustino Pérez Hernández Hospital (Institutional Review board) and by the Institutional Review Board from the Provincial Centre of Hygiene, Epidemiology and Microbiology, Matanzas- Cuba. The enrollment also required that the agreement model was signed by one of the child’s parents or legal guardians, after being fully informed about the aim of the study. Instructions on how to collect the faeces were also provided in writing to the parents or legal guardians of the children.

## Results

A total of 104 children were included, 55 (52.9%) male and 49 (47.1%) female. Surprisingly, 74 (71.1%) children were harboring some species of IP and 47 (45.2%) were poly-parasitized. No statistically significant difference was observed among presence of IP and sex and also age (P>0.05).

*Giardia duodenalis* and *Blastocystis sp.* were the most common parasites found, with prevalence rates of 54.8% (57 cases) and 38.5% (40 cases), respectively. Only 2 (1.9%) cases were infected by *Entamoeba histolytica/E. dispar* complex. The nonpathogenic *Entamoeba coli* (3/2.9%) and *Endolimax nana* (8/7.7%) were also notified. The overall prevalence of Soil transmitted Helminthes (STH) infections was 12 (11.5%) with *Ascaris lumbricoides* (7/6.7%) being the most predominant, followed by *Trichuris trichiura* (3/2.9%), and *Strongyloides stercoralis* (1/1%). *Enterobius vermicularis* (2/1.9%) and *Taenia sp.* (1/1%) were also identified (Table 1). Concerning the intensities of STH infections, only light infections of *Ascaris lumbricoides* and *Trichuris trichiura* were found.

**Table 1:** Intestinal parasite infections diagnosed by faecal examination from 104 children attending the day care centre in Matanzas city, Cuba.

Parasite species	No. %	(CI 95%)
Protozoa		
*Giardia duodenalis*	57	54.8 (45.2–64.4)
*Blastocystis sp.*	40	38.5 (29.1–47.8)
*Endolimax nana*	8	7.7 (2.6–12.8)
*Entamoeba coli*	3	2.9 (−0.3–6.1)
*Entamoeba histolytica/E. dispar*	2	1.9 (−0.7–4.6)
Helminths		
*Ascaris lumbricoides*	7	6.7 (1.9–11.5)
*Trichuris trichiura*	3	2.9 (−0.3–6.1)
*Enterobius vermicularis*	2	1.9 (−0.7–4.6)
*Strongyloides stercoralis*	1	1.1 (−1.8–2.8)
*Taenia sp.*	1	1.1 (−1.8–2.8)

Drinking untreated water [RR = 22.60; CI (5.83–87.71)], eating fruits without washing [RR = 8.40; CI (3.69–19.12)], and sucking fingers and/or nail biting [RR = 8.75; CI (1.35–56.88)] were the risk factors significantly associated to IP (Table 2). Of those bad habits, sucking fingers and/or nail biting was identified in all infected children. Drinking untreated water and eating fruits without washing were identified in 77% and 84% of infected individuals respectively.

**Table 2:** Significant relation for the intestinal protozoa and STH in the study population.

Inadequate Personal hygienic habits		Infected by protozoa or STH	
		N = 74	
Characteristics	No. (%)	No. (%)	RR (CI 95%)
Drinking untreated water	58		
	(55.8%)	(98.3%)	(5.83- 87.71) *
Eating fruits without washing	62	62	8.40
	(59.6%)	(100%)	(3.69- 19.12) *
Sucking fingers and/or nail biting	93	74	8.75
	(89.4%)	(79.6%)	(1.35- 56.88) *

Note. RR = Relative risk; CI = Confidence interval;

* Significant.

## Discussion

Although intestinal parasitic infections among children remain a global issue, the current information on such infections in children attending day care centres in Matanzas, Cuba is very limited [7]. It is known that the transmission of enteroparasites depends on the presence of infected individuals, sanitation deficiencies and, principally, the socioeconomic and cultural conditions of the population [1].

In the present study the prevalence of IP was found to be high being protozoan parasites *Giardia duodenalis* and *Blastocystis sp*. the most represented species. Some factors need to be considered in order to understand this result. First, it is well known that intestinal parasites may be more easily identified when the number of faecal samples from each individual is increased and when different coproparasitological techniques, especially concentration methods, are employed [10]–[11]. Second; children attending day care centres have not well established the adequate hygiene habits and third, because of the convergence of many individuals in a reduced area the risk to disseminate intestinal parasitic infections is very high [3], [12].

Another dimension of the problem has been highlighted by Escobedo and colleagues [13] in an interesting paper about caregiver perspectives for the prevention, diagnosis and treatment of childhood giardiasis in Havana- Cuba. This study demonstrated the existence of myths and misconceptions about *Giardia duodenalis* infection. The same study identified as the most commonly mentioned reasons for not adopting preventive behaviors the lack of time due to outdoor activities and the limitation of combustible distribution. Despite the knowledge widely present about the actions to control IP relatives do not play complete attention to those activities. Probably these results could be extra poled to others parasitic infections found in day care sites and clarify why they are still present despite all effort made by doctors and educators.

One study, carried out among preschool children from Brazil [3], showed that 29.3% of them were infected by IP and 6.7% presented polyparasitism. Like to our study *Giardia duodenalis* was the most frequent parasite identified. The general prevalence of IP, less than those found in this study probably could be associated with the techniques used. In Pakistan [14], another study was carried out to estimate prevalence and identify factors associated with intestinal parasitic infections among 1 to 5 years old children. The general prevalence of 52.8% again demonstrates the frequency of IP in these institutions. Similar to the first mentioned works, *Giardia duodenalis* was the commonest IP identified.

*Blastocystis* is a unicellular organism of controversial clinical significance often identified in stools from patients worldwide [15]–[17]. Considering that transmission of *Blastocystis* occurs by fecal-oral spread this parasite needs to be following closer in day care sites. In the present study, 38.5% of children studied were positive to *Blastocystis sp*. STH infections were less notified. A possible explanation could rise from the place where the institution is located. Because of limited contact with soil, children have less opportunity to be in contact with the infective stages of STH. A previous study in Matanzas, Cuba also corroborates this observation [7].

*Ascaris lumbricoides* was the STH most identified and probably the commonest intestinal worm in this city considering the opinion of experienced epidemiologist (unpublished data). Vegetables or fruits could be the source of infection in this site considering those facts exposed by Escobedo and colleagues [13] and the generalized bad habits of eating fruits without proper washing.

The low occurrence of *Enterobius vermicularis* in this day care centre may be associated with the use of a nonspecific method for diagnosis. Few studies concerning the frequency of IP in children identified *Enterobius vermicularis* eggs by the Graham method (anal swab) [18], so the true prevalence of this worm could be underestimated.

Like in other studies; drinking untreated water, eating fruits without washing, and sucking fingers and/or nail biting were risk factors statistically associated with the presence of IP [3], [4], [9], [14]. These bat habits are largely recognized as risk factors for IP worldwide [19], [20] primarily in rural areas and poor urban areas of low-income and middle-income countries.

In Berlin, Germany, Sagebiel agree with our results and highlights the need of additional education and training on proper toilet and food hygiene to reduce the possibility of child-to-child transmission [21]. It is very interested the case control study published by Gurgel and colleague in Brazil in 2005 [22] where found that attending day care centers is related to intestinal parasites and the risk of infections is 1.5 times higher in children who are on that sites compared with those no related with that institutions. Another case control study, this carried out in Havana- Cuba [23], also demonstrate the importance of eating fruits without washing as a risk factor to be infected by IP, in this case *Giardia duodenalis*.

The present study demonstrates the importance of periodical surveys in day care centres considering IP are highly prevalent in this setting. This kind of research could be the way to identify infected children and prevent with their treatment, educational activities, and follow up visits the possible negative effect of IP on their grown and development. A day care free of IP is also desirable to increase the quality of live in communities both, rural and urban all over the world.

### Conclusions

Intestinal parasitic infections by *Giardia duodenalis* and *Blastocystis sp.* are highly prevalent in the day care centre under study. Given that both IP are intimately associated poor environmental sanitation and lack of clean water supply, it is crucial that these factors are addressed effectively. With effective control measures in place, this day care centre will have a greater opportunity for a better future in terms of health and educational achievement so an educational intervention is running today.
